# The effect of mindfulness training on extinction retention

**DOI:** 10.1038/s41598-019-56167-7

**Published:** 2019-12-27

**Authors:** Johannes Björkstrand, Daniela Schiller, Jian Li, Per Davidson, Jörgen Rosén, Johan Mårtensson, Ulrich Kirk

**Affiliations:** 1 Department of Psychology, University of Southern Denmark, Odense, Denmark; 2 Department of Psychology, Uppsala University, Uppsala, Sweden; 3 Department of Psychology, Lund University, Lund, Sweden; 4 Departments of Psychiatry and Neuroscience, and Friedman Brain Institute, Icahn School of Medicine at Mount Sinai, New York, NY USA; 5 School of Psychological and Cognitive Sciences and Beijing Key Laboratory of Behavior and Mental Health, Peking University, Beijing, China; 6 Department of Clinical Sciences, Lund University, Lund, Sweden

**Keywords:** Human behaviour, Translational research

## Abstract

Anxiety and trauma related disorders are highly prevalent, causing suffering and high costs for society. Current treatment strategies, although effective, only show moderate effect-sizes when compared to adequate control groups demonstrating a need to develop new forms of treatment or optimize existing ones. In order to achieve this, an increased understanding of what mechanisms are involved is needed. An emerging literature indicates that mindfulness training (MFT) can be used to treat fear and anxiety related disorders, but the treatment mechanisms are unclear. One hypothesis, largely based on findings from neuroimaging studies, states that MFT may improve extinction retention, but this has not been demonstrated empirically. To investigate this question healthy subjects either completed a 4-week MFT- intervention delivered through a smart-phone app (n = 14) or were assigned to a waitlist (n = 15). Subsequently, subjects participated in a two-day experimental protocol using pavlovian aversive conditioning, evaluating acquisition and extinction of threat-related responses on day 1, and extinction retention on day 2. Results showed that the MFT group displayed reduced spontaneous recovery of threat related arousal responses, as compared to the waitlist control group, on day 2. MFT did not however, have an effect on either the acquisition or extinction of conditioned responses day 1. This clarifies the positive effect of MFT on emotional functioning and could have implications for the treatment of anxiety and trauma related disorders.

## Introduction

Anxiety and trauma related disorders are a major public health concern, being one of the leading causes of disability in global burden of disease estimates^1^. Their etiology is believed to depend on threat related associative learning, where otherwise neutral cues and contexts become associated with aversive experiences^2^. Although fear learning is adaptive, increasing the organism’s ability to predict and respond to threats in the environment, it can also lead to psychopathology. In humans, when fear reactions and avoidance behavior become excessive this may result in pathological anxiety. This type of learning has been studied extensively in animals and humans using pavlovian fear/threat conditioning^3,4^. In this procedure, neutral cues, typically tones or pictures, are paired with an aversive unconditioned stimulus (US), such as a painful electrical shock^5^. After repeated pairings the previously neutral cues start to elicit defensive responses, such as freezing behavior, increased physiological arousal and avoidance, thus becoming a conditioned stimulus (CS). This type of learning depends on a distributed neural circuit involving separate sensory input pathways for the CS and US respectively, converging in the amygdala, where the fear memory is formed, and subsequently controls CS elicited fear expression^6^. In humans, neuroimaging studies have found that fear conditioning leads to increased activity in a distributed network including the amygdala, anterior insula, dorsal anterior cingulate cortex and dorsal parts of the midbrain, and that activity in several of these regions predicts the magnitude of conditioned responses^3,7^.

Cognitive behavioral therapy (CBT), often including exposure based strategies, has been shown to be effective in treating anxiety and trauma related disorders, but not everyone responds to these treatments, controlled effect-sizes are generally found to be in the small to medium range, leaving room for improvement^8–14^, and the quality of the empirical evidence has been questioned^15^. As a consequence, there is a need to develop new forms of treatments for these disorders, or optimize existing ones. The theoretical basis of exposure therapy is extinction learning^2,16^ where the CS is repeatedly presented without the aversive outcome. Extinction typically leads to a reduction in threat responses but does not alter the original CS-US association, as evident by the fact that conditioned responses tend to return with the passage of time or when the CS is presented in a novel context^17,18^. Instead, extinction is thought to depend on new learning, resulting in the formation of a safety memory that subsequently suppresses fear expression to original cues^17^. Based on studies in animals^19^ and humans^3,20–23^, this type of learning is held to be dependent on the ventromedial prefrontal cortex (vmPFC) and the hippocampus, regions that through amygdala connections suppress fear expression following extinction. Notably, research in clinical populations have shown that both anxiety and trauma related disorders are characterized by deficiencies in extinction learning and recall, as well as abnormal activation patterns in associated neural structures^21,24–28^, indicating that this could be a contributing underlying cause for these disorders. Consequently, it has been argued that basic research on extinction learning may be translated to clinical exposure-based interventions to improve outcomes^18,25^. A lot of this research has been centered on the question of how to improve extinction retention which, if translated successfully, could have a positive impact on immediate outcomes of clinical interventions and reduce the risk of relapse^16,18,29–31^. As has been suggested in several recent reviews^32–34^, one way to accomplish this could be through the use of mindfulness training (MFT).

**Figure 1:**
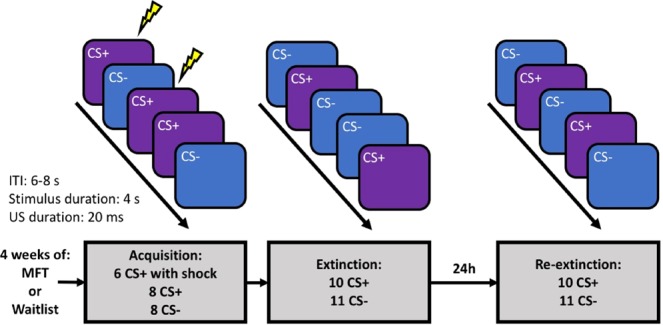
Experimental design: Subjects were randomized to either 4-weeks of daily MFT or a waitlist condition and subsequently completed a 2-day fear and extinction learning protocol. During acquisition, two neutral cues (a blue or purple square) were repeatedly presented on a computer screen, and one of the cues (CS+) was paired with an aversive electrical shock (US) with a reinforcement rate of 43%, whereas the other cue (CS−) was never paired with a shock. Acquisition always began with a reinforced CS+ presentation and subsequent stimulus presentations were pseudorandomized, so that none of the stimuli appeared more than two times consecutively. Then the subjects underwent immediate extinction where the CS+ and CS− were repeatedly presented but the US was omitted. To evaluate extinction retention, subjects returned to the lab the next day and the extinction procedure was repeated. Extinction and re-extinction always began with a CS− presentation, and consequently involved one extra presentation of CS−, subsequent stimulus presentations were pseudorandomized. Across all phases, stimuli were presented for 4 s, with a 6–8 s inter-trial interval (ITI). The strength of the US was set individually for each subject and had a duration of 20 ms.

An emerging literature indicates that mindfulness-based interventions can be used to treat a range of psychological problems, including anxiety and trauma-related disorders^8,35–37^, but the treatment mechanism is so far unclear. It has been argued that MFT exerts its beneficial effects on psychological functioning by increasing attention and emotion regulation, and more specifically that mindfulness may have a positive impact on extinction learning and retention of extinction memories^33,34,38^. The basis for this hypothesis rests on findings from neuroimaging studies, showing that MFT affects both functional and structural aspects of brain regions linked to fear and extinction learning^33^. Previous studies have fairly consistently shown that MFT reduces amygdala activity and volume, and increases activity and volume of the hippocampus^32^. Furthermore, MFT is associated with increased activity in the PFC, and altered functional connectivity between the PFC and the amygdala^32,33^. Similarly, a recent study found that structural connectivity between the vmPFC and the amygdala was increased after MFT^38^. Because reduced fear expression following extinction training is believed to be dependent on hippocampal and prefrontal regulation of fear expression controlled by the amygdala^19,20^, this provides clear implications for a possible role of MFT to enhance extinction retention. One previous study^38^ also found indications that MFT has a positive effect on extinction retention using an experimental fear and extinction learning protocol. However, pre-intervention group differences in that study could account for the experimental effects, rendering the results inconclusive. Thus, there is as of yet no direct evidence that MFT improves extinction retention. Here we aimed to answer this question using a between group design where healthy subjects were randomly assigned to receive either a 4-week smart-phone delivered MFT-program (n = 14) or to a wait-list condition (n = 15), and subsequently completed a 2-day experimental protocol consisting of aversive pavlovian conditioning and immediate extinction on day 1, followed by an extinction retention test 24 hrs later on day 2, see Fig. 1.

## Methods

### Participants

Participants were recruited through public advertisements posted in public areas around Lund University campus, and were randomly assigned to either 4 weeks of mindfulness training (n = 14) or to a waitlist control group (n = 15). Due to equipment failure, data from three subjects were lost for the fear acquisition phase and data from two subjects were lost for the extinction and re-extinction phase, all in the MFT-group. Thus, 26 subjects (MFT; n = 11; Waitlist; n = 15) where available for analysis of conditioned threat-responses. The study was approved by the Swedish Ethical Review Authority, Regional Review Board in Lund, all procedures were conducted in accordance with local guidelines and regulations, and all subjects provided written informed consent. Apart from gaining access to the mindfulness app used in the intervention, subjects did not receive any compensation for participating. All healthy participants between 18–65 were eligible for inclusion. The collected sample had an average age of 35.1 ± 6.2 (mean ± SD) years, and was highly similar in both groups, 35.6 ± 5.8 years in the MFT group and 34.5 ± 6.5 years in the control group (t = 0.48; df = 27; p = 0.64). Participants of both genders were eligible for inclusion but the collected sample ended up being primarily female (79.3% female), with similar gender distribution in the MFT group (85.7% female) and control group (73.3%). All subjects were university employees and thus had a high level of educational attainment exceeding 12 years for all subjects. In order to be included in the study, the subjects were required to be in general good health, have a good understanding of written and spoken English (the intervention was delivered in English) and have access to a smart-phone. Extensive previous experience of mindfulness or other meditation practice, presence of self-reported psychiatric condition or ongoing treatment for a psychiatric condition were exclusion criteria. Psychological health status was also assessed with structured self-report questionnaires, the Beck Anxiety Inventory (BAI) and Beck Depression Inventory (BDI), before and after the intervention^39,40^. To verify that groups did not differ in dispositional mindfulness prior to treatment, and also as a manipulation check, subjects completed the Mindfulness Attention Awareness Scale (MAAS)^41^, before and after the intervention.

### Procedures

#### Mindfulness intervention

The intervention was delivered through a commercially available smart-phone app (Headspace; https://www.headspace.com) that has been used by our group^42^ and others^43–45^ in previous intervention studies. The content of the training was based on well-established concepts and practices within the mindfulness literature^46^ and entailed short (10–20 minutes) daily practice in guided mindfulness meditation, with instructions delivered through short animated videos and sound files in the app. Similar to a previous intervention study^42^, the subjects did not receive any prior training in mindfulness but were simply given access to the app and instructed to engage in training as presented. The training program involved increasing amounts of daily meditation practice, where 10 min/day was the target amount for the first 10 days, increasing to 15 min/day for the following 10 days, and 20 min/day for the remainder of the program. By examining user data provided by app developers on how much time each subject had spent meditating with the app, we could confirm that all participants showed acceptable adherence to the program. Participants were informed of this and consented to us gaining access to their user data before entering the study.

#### Aversive pavlovian conditioning and extinction procedure

After completing the intervention or waitlist period, subjects came to the lab on two consecutive days to undergo fear acquisition followed by immediate extinction (day 1), and re-extinction (day 2), see Fig. 1. They were sat in front of a computer screen were experimental stimuli were displayed. The experiment was implemented using E-prime 2.0 (Psychology Software Tools, Pittsburgh, PA). To evaluate fear acquisition, extinction learning and extinction retention, even-related skin conductance responses (SCRs) were measured during all experimental phases using two Ag-AgCl electrodes attached to the palmar surface of the index and middle finger of the non-dominant hand. SCRs were amplified and recorded with a sampling rate of 1000 Hz using the MP150 Biopac system and Acqknowledge software version 4.2 (Biopac Systems). The aversive US consisted of an electrical shock with a 20 ms duration delivered to the back of the ring finger of the non-dominant hand using a Grass SD 9 stimulator (Grass-Telefactor). Before the experiment started, the chock level was set individually for each subject by delivering electrical shocks of increasing strength until a level that the subject perceived as “uncomfortable but not painful” was reached. Due to a clerical error information on the individual shock level for each subject were not saved, and this data is therefore not presented. The fear and extinction protocol was adapted from a previous study^47^, with the exemption that extinction followed immediately after fear acquisition. During fear acquisition two neutral cues (a blue and a purple square on a black background), where repeatedly presented in pseudo randomized order, where one of the cues (CS+) was repeatedly paired with the US, whereas the other cue (CS−) was not. The CS+ was presented 14 times, 6 of which were reinforced trials were the CS+ co-terminated with US. The CS− was similarly presented 8 times but never paired with the US. Acquisition always began with a reinforced CS+ presentation, and subsequent stimulus presentations were pseudorandomized, with reinforced CS+ presentations evenly distributed across the learning protocol. Extinction followed immediately after acquisition, where the CS+ was presented 10 times and the CS− was presented 11 times. No US was delivered during this phase, although the subjects where still attached to the shock apparatus. The next day, subjects returned to the lab and underwent re-extinction, where the extinction procedure was repeated using the same parameters. Extinction and re-extinction always began with a CS− presentation and consequently involved one additional presentation of the CS−. The subsequent stimulus presentations were pseudorandomized. Two pseudorandomized trial orders were used, so that no stimulus of the same kind occurred more than two times consecutively and the two trial orders were counterbalanced across participants. During all phases, CS+ and CS− stimuli were presented for 4 seconds on each trial, with an inter-trial interval (ITI) ranging between 6–8 seconds.

### Preprocessing and analysis

First the quality of skin conductance data was assessed through visual inspection. Data from three subjects was lost for the acquisition phase and data from two subjects were lost for the extinction and re-extinction phase, due to electrode malfunction. Thus, the final sample size was n = 11 for the MFT group and n = 15 for the control group, for analyses evaluating SCRs. Subsequently, a low-pass filter of 0.5 Hz and a high-pass filter of 0.05 Hz was applied to the skin conductance signal in order to filter out high frequency disturbances and to reduce baseline drift. Then, similar to previous studies^48–51^, event-related SCRs were extracted, where the mean value of the skin conductance signal 1.2–1.5 seconds after stimulus onset was deducted from the peak value during 1.5–4.5 seconds after stimulus onset. To reduce between subject variability SCRs were then range-corrected by dividing each response by the largest CS elicited SCR across all experimental phases^52^. CS difference scores were then calculated by subtracting the CS− elicited SCR from the CS+ elicited SCR on a trial by trial basis. For acquisition, only the non-reinforced CS+ presentations were used for CS difference score calculations, and reinforced CS+ presentations were omitted from analysis. For extinction and re-extinction, the last CS− presentation was omitted from analysis. Averaged CS difference scores were used for analysis of acquisition and extinction respectively. Spontaneous recovery was defined as increases in CS difference scores from the end of extinction to the beginning of re-extinction similar to previous studies^48–51,53^, and to provide a single measure for extinction retention we calculated a spontaneous recovery index (CS difference score for the last trial of extinction deducted from the first trial of re-extinction). All statistical analyses were implemented in JASP (Version 0.9). All data are available at request made to the corresponding author.

## Results

### Self-report questionnaires and manipulation checks

To verify that subjects were psychologically healthy their self-reported levels of anxiety and depression where investigated using the BAI and BDI. Since subjects reported they had no on-going psychopathology at inclusion we expected low scores for these measures and did not expect to see any effect of the intervention. The Shapiro-Wilks test confirmed that the data did not violate assumptions of normality, see Table S1, and consequently we performed parametrical test. Independent t-tests for the pre- and post-intervention assessments, as well as the change-score (pre-score deducted from post-score) for the BAI and BDI respectively, indicated that the groups were similar as to self-reported depression and anxiety symptoms both before and after the intervention, as well as change from before to after treatment (t < 1.1; p = n.s. for all comparisons, see Table S2). The group averages were in the lower end of the mild range for BAI, and well within the minimal range for BDI^39,40^ confirming that the sample did not show signs of anxiety or depression related psychopathology. Descriptive statistics for these measures are presented in Table S3.

**Figure 2:**
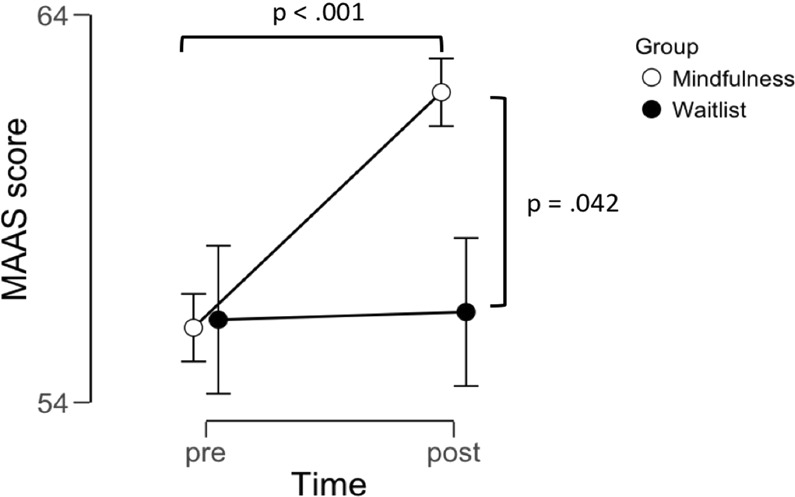
The effect of MFT on self-rated dispositional mindfulness. As a manipulation check for the MFT intervention we evaluated the effect of the training program on dispositional mindfulness using the validated questionnaire MAAS as an outcome measure. As expected, the MFT group showed significant increases on this measure whereas no change was observed in the control group. No group differences were present prior to the intervention but the MFT group scored significantly higher than the control group post intervention. Displayed p-values were obtained using paired t-test and independent t-test (single tailed). Points and error-bars denote mean and SEM.

In order to investigate adherence to the training program in the MFT group, user data on how many minutes subjects had engaged in training each day were obtained from the app developer. Logged user-data confirmed that all subjects showed acceptable adherence to the intervention. Daily training duration across all subjects in the MFT group was 13.2 ± 1.7 minutes (mean ± SD), with a range of 10.0–15.4 minutes (min-max). Furthermore, to verify that groups did not differ in dispositional mindfulness prior to treatment, and also as a manipulation check, subjects completed the MAAS^41^, before and after the intervention. Assumptions of normality were confirmed using the Shapiro-Wilks test (see Table S1), and parametrical analysis was used. An independent t-test showed that groups reported similar levels of dispositional mindfulness prior to treatment (t = −0.05; df = 27; p = 0.961). To verify the effect of the intervention we performed the same analysis for the post-measurement and change-score (pre-score deducted from post-score). Because we had an a priori hypothesis that the intervention group would increase in this measure but the control group would not^42^ we used single-tailed t-tests for these analyses. Independent t-test showed significant group differences both for the post-score (t = 1.78; df = 27; p = 0.042, single-tailed) and the change score (t = 1.93; df = 27; p = 0.032, single tailed). Paired t- tests between the pre- and post-tests in each group respectively confirmed that the intervention group showed significant increases (t = 4.92; df = 13; p > 0.001) but no change in the control group (t = 0.0.07; df = 14; p = 0.942), see Fig. 2. Descriptive statistics for this measure are presented in Table S3.

### Acquisition and extinction of conditioned responses

**Figure 3:**
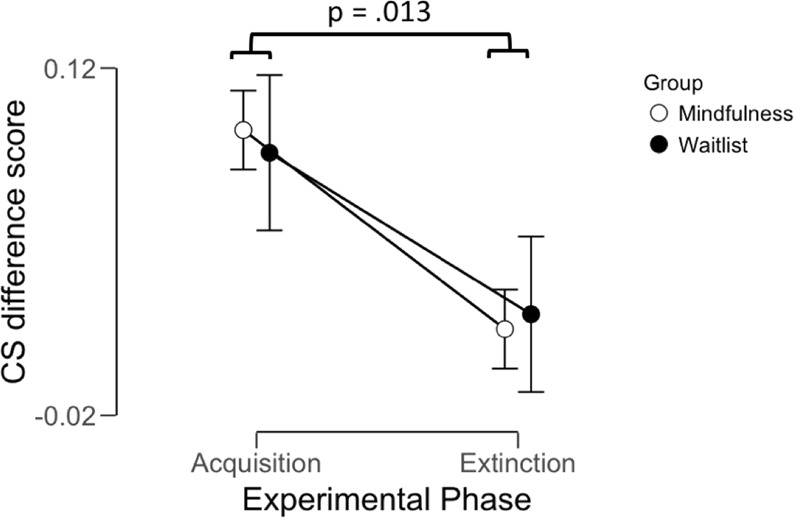
Threat related arousal responses during acquisition and extinction of pavlovian aversive conditioning on day 1. Analysis of average CS difference scores for the acquisition and extinction phase day 1 demonstrated successful acquisition and extinction of conditioned threat responses across the entire sample. Scores during acquisition were significantly different from zero, demonstrating fear learning, and scores during extinction were significantly lower compared to acquisition, demonstrating extinction learning. No group differences in threat related arousal responses were observed during either phase, nor in decreases from acquisition to extinction. Displayed p-value were obtained through pairwise t-test. Points and error bars denote means and SEMs.

To evaluate acquisition and extinction of conditioned threat responses, CS difference scores were averaged over the entire acquisition and extinction phase respectively, and the Shapiro-Wilks test confirmed that assumptions of normality were met, see Table S1. Descriptive statistics for all SCR derived outcome measures are presented in Table S4. Pertaining to acquisition, a one- sample t-test confirmed that conditioned responses were successfully acquired, showing a significant difference from zero (t = 4.33; df = 25; p < 0.001). Subsequently, to evaluate extinction learning, as well as potential group differences during fear and extinction learning, CS difference scores averaged over the extinction phase and together with the acquisition score, were entered into a 2 × 2 repeated measures ANOVA with factors Phase (Acquisition; Extinction) and Group (Waitlist; Mindfulness). The results demonstrated that fear responses were successfully extinguished reflected in a significant main effect of Phase (F = 7.08; p = .013), see Fig. 3. Post-hoc t-tests confirmed that CS difference scores were larger during acquisition compared to extinction (t = 2.66; df = 25; p = .013), and reactions during extinction were not significantly different from zero (t = 1.36; df = 25; p = .186). No group differences were identified as both the main effect of Group (F = 0.01; p = .941) and the Phase x Group interaction (F = 0.75; p = .787) were non-significant. Similarly, t-tests for each phase separately did not show any group differences either during the acquisition (t = 0.21; df = 24; p = .832) or extinction (t = −0.42; df = 24; p = .680). Trial by trial analysis of each phase separately yielded similar results, see Fig. S1 in the Supplemental material.

### Extinction retention

**Figure 4:**
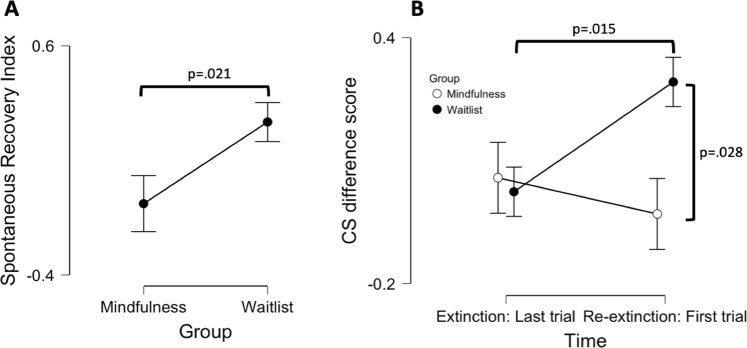
Mindfulness training enhances extinction retention. (**A**) In order to evaluate between-group differences in extinction retention a spontaneous recovery index (SRI) was calculated by deducting the CS elicited threat response at the last trial of extinction from the first trial of re-extinction. An independent t-test confirmed a significant group difference on the SRI where the control group (n = 15) showed clear indications of recovery whereas no recovery was observed in subjects that had undergone MFT (n = 11). (**B**) This effect was driven by differential responding to the conditioned stimuli at the beginning of the re-extinction phase rather than the end of the extinction phase. A significant between group difference in magnitude of threat responses was observed during the first trial of re-extinction but not during the last trial of extinction, and the control group, but not the mindfulness group, showed significant increases in threat responses from the end of extinction to the beginning of re-extinction. Displayed p-values were obtained through Wilcoxon Signed-Rank Test and independent t-test respectively. Points and error bars denote means and SEMs.

To evaluate the main hypothesis that MFT improves extinction retention, reflected in reduced spontaneous recovery, we calculated a spontaneous recovery index (SRI) where CS-difference scores during the last trial of extinction were subtracted from CS-difference scores during the first trial of re-extinction, similar to previous studies^50,51,53,54^. The Shapiro-Wilks test confirmed that assumptions of normality where met (see Table S1) and group differences were evaluated with an independent t-test. In line with the hypothesis, the result showed a significant group difference and a large effect-size (t = 2.47; df = 24; p = .021; Cohen’s d = 0.98), see Fig. 4A. One-sample t-tests for each group separately showed that spontaneous recovery could be confirmed only in the waitlist group reflected in an SRI significantly higher than zero (t = 3.15; df = 14 p = .007), but not in the mindfulness group (t = −0.72; df = 10; p = .487). To evaluate whether strength of initial fear learning influenced these results, we used an ANCOVA with SRI as the dependent variable, Group (Mindfulness; Waitlist) as a fixed factor and then added the average CS difference scores during acquisition as a covariate. This did not influence the results, with the analysis still showing a significant effect of Group (F = 4.48; p = .046; η^2^ = 0.16). Similarly, adding average CS difference score during extinction as a covariate did not influence the results either, still with a significant effect of Group (F = 5.91; p = .023; η^2^ = 0.20), thus the effect was not influenced by either the degree of initial acquisition or extinction of conditioned responses.

For further clarification we performed follow-up analyses investigating whether the effect was driven mainly by differential responding to the conditioned stimuli at the beginning of re-extinction or during the end of the extinction phase. Thus, we performed between and within group analysis for the last trial of extinction and the first trial of re-extinction separately. The Shapiro-Wilks test indicated that assumptions of normality were only met for the first-trial of re-extinction, but not the last trail of extinction (see Table S1), and parametrical and non-parametrical tests were used accordingly. The results indicated that the effect was mainly driven by differential responding to the conditioned stimuli during the first trial of re-extinction where an independent t-test showed a significant group difference with a large effect size (t = 2.35; df = 24; p = .028; Cohen’s d = 0.93), whereas no group differences where observed for the last trial of extinction (U = 78.50; p = .856) as indicated by a Mann-Whitney U test, see Fig. 4B. Within group analysis for each group separately confirmed significant differential responding to the conditioned stimuli only in the waitlist group during the first trial of re-extinction where a one-sample t-test showed a significant difference from zero (t = 4.17; df = 14; p < 0.001), but not in the MFT group (t = −0.23; df = 10; p = .821), whereas during the last trial of extinction neither the waitlist group (Z = 75.00; p = .167) nor the MFT group (Z = 39.00; p = .262) showed differential responding to the conditioned stimuli, as indicated by the Wilcoxon Signed-Ranks Test. Furthermore, significant increases in CS difference score from the end of extinction to the beginning of re-extinction could only be confirmed in the waitlist group (Z = 18.00; p = .015) but not in the MFT group (Z = 30.00; p = .831) using the Wilcoxon Signed-Ranks Test, see Fig. 4B. Taken together the results indicate that the two groups achieved adequate extinction on day 1 where they did not differ from each other, and that group differences on the SRI were driven by differential responding during the beginning of re-extinction day 2, supporting the conclusion that MFT has a specific effect on extinction retention. Trial by trial analysis of the entire re-extinction phase separately, showed similar results, see Fig S1 in the Supplemental Material.

## Discussion

The results showed that brief MFT improves the retention of extinction learning when compared to a waitlist control group. This was reflected in an absence of spontaneous recovery of conditioned threat responses in the intervention group 24 hrs after extinction training, whereas the control group showed clear indications of recovery. No group differences were found during either acquisition or extinction of conditioned responses on day 1, nor did individual differences in the strength of conditioned responses during acquisition or extinction influence the effect on extinction retention. This indicates that MFT does not have a generally dampening effect on threat related arousal responses, but rather a specific effect on retention of safety learning. The results supports and clarifies the hypothesized positive impact of MFT on extinction learning that has been previously postulated^32–34^, but up until now has lacked support in the empirical literature.

Although this study was performed on healthy individuals, the results could have implications for research on clinical populations and exposure-based treatments of psychopathology^16,18,31^. The findings may partly explain the clinical efficacy of mindfulness interventions for anxiety and trauma related disorders. Considering that extinction learning and recall has been found to be impaired in populations with these disorders^24–28^, improved extinction retention afforded by MFT may enhance the effects of naturally occurring exposure to anxiety eliciting cues and contexts that these patients encounter in their day to day lives, which in turn could cause reduction in anxiety symptoms. Furthermore, because vmPFC hypoactivation during extinction recall has been demonstrated in patients with anxiety and trauma related disorders^21,27^, MFT may achieve its effects on extinction retention by normalizing functioning of prefrontal regions that suppress the expression of fear responses, as previously stipulated. The findings also suggest that MFT may not only be used as a stand-alone treatment intervention for anxiety and trauma related disorders, but might be employed as an add-on to exposure-based treatment interventions. Delivering low-intensity MFT prior to exposure therapy, thereby priming the patient for extinction learning, could improve both immediate outcomes through reduced between-session recovery of fear responses as well as reduce the risk of relapse. Similarly, the findings suggest that MFT could be used as a prevention strategy to mitigate the risk of trauma and anxiety related problems in high-risk groups. However, further studies investigating these issues in populations with ongoing psychopathology are needed in order for the clinical implications of the current findings to be firmly established.

The effect of MFT on extinction retention has to our knowledge only been investigated in one previous study^38^, where subjects were randomized to either an 8-week course of mindfulness-based stress reduction (MBSR) or a wait-list control group, and completed a 2-day fear and extinction protocol similar to ours, both before and after the intervention. In line with our results they found that extinction retention improved in the MBSR group from the pre- to post-test, whereas no change was observed in the control group. However, because significant pre- intervention between group differences on extinction retention were present, no firm conclusions could be made regarding the effect of MBSR on this outcome measure. Similar to our study they did not find any indications for group differences pertaining to extinction learning on day 1. However, they did find a significant between-group effect for fear acquisition. In the control group, they found that discrimination between CS+ and CS− decreased from the pre- to post-test, but the MBSR group maintained high discrimination both before and after the intervention, whereas we did not find any between group differences during fear acquisition. A possible reason for this discrepancy could be that Hölzel and colleagues used a pre-post design and that the effect that they found for acquisition appears to be an interaction effect of MBSR training and repeated testing of fear conditioning. Because we wanted to avoid any effects of previous testing on the outcome measures of interest, and wanted the subjects to be naïve as to the experimental protocols, we used a single post-test design which could explain the absence of any effects on fear acquisition in our study.

Although the results clearly demonstrate that MFT has a positive impact on extinction retention, there are limitations indicating that the findings should be interpreted with caution. The main limitation is the small sample size, that was restricted due to difficulties in recruiting eligible participants willing to engage in daily MFT for four weeks, and the unfortunate loss of data only in the intervention group. The only previous study investigating the effect of MFT on extinction retention^38^, reported a large effect size of Cohen’s d = 1.15 (converted from η^2^ = 0.25). Using this effect size to perform sample size calculation^55,56^ shows that to obtain a statistical power level of 0.8 and an alpha level of 0.05 a sample size of n = 11 in each group is necessary for a single tailed hypothesis and, n = 13 for a two-tailed hypothesis, indicating that the study is somewhat underpowered. Similar studies using larger samples should be performed in order to investigate the replicability of our findings. In relation to the characteristic of our sample, the objective of this study was to investigate the research question in healthy controls. To this end, the absence of psychopathology was ascertained through self-report, and further verified using the BDI and BAI which confirmed the absence of self-reported anxiety and depression related problems. We did not, however, collect information on the participants’ previous exposure to trauma. Given that we discuss our findings in relation to trauma related psychopathology, this could be relevant information that may influence the results, and should be further examined in future studies. Furthermore, due to a clerical error, information on the individually set shock level were not saved. Consequently, we were unable to evaluate potential group differences for this variable, which could have influenced the results. Considering that MFT could have an effect on the shock levels subjects chose, this is a possible confound and should investigated in future studies. In relation to experimental design, we used a protocol involving immediate extinction, not allowing for consolidation of the previously acquired fear memory. This may limit the applicability of the findings to clinical interventions, in that exposure-based treatments for anxiety and trauma related disorders are aimed at reducing the negative impact of fear memories that have been established months or years prior to treatment. Future studies could make use of 3-day designs, were acquisition and extinction are separated by 24 hrs or more, investigating whether the present findings also extend to extinction of consolidated fear memories. Additionally, because we did not collect any neuroimaging data in this study, we can only speculate as to the underlying neural substrates that are hypothesized to underlie the observed effects. Based on previous neuroimaging studies of MFT^32^, the effects could be due to alterations directly in the amygdala with subsequent effects on fear expression, or due to alterations in regions that are known to exert top-down control over the amygdala driven fear expression subsequent to extinction learning, such as the hippocampus and the vmPFC. Considering we only observed group differences for spontaneous recovery and not during the preceding experimental phases, the latter seems to be the more likely hypothesis, but this remains to be empirically tested. Future studies should incorporate online measurements of brain activity during the experimental procedures in order to evaluate this question.

In conclusion, in this study we show that fairly brief MFT can significantly reduce spontaneous recovery of previously extinguished threat-related arousal responses, an effect that has been hypothesized in several previous reviews^32–34^, but never empirically demonstrated. However, due to the small sample size the results should be considered as preliminary until replicated in larger studies. Nevertheless, the findings provide some clarifications regarding the positive impact of MFT on emotional functioning and has implications for the treatment of trauma and anxiety related disorders, where MFT may be used as an augmentation strategy for existing exposure-based protocols. Previous neuroimaging studies suggest that the observed effect may well be mediated through structural and functional alterations to brain regions related to safety learning, which remains to be investigated in future studies.

## Supplementary information

Supplementary material

## Data Availability

The datasets generated and analyzed during the current study are available from the corresponding author on reasonable request.
