# Supplementary material for: The effect of mindfulness training on extinction retention
Source: Sci Rep. 2019 Dec 27;9:19896. doi: 10.1038/s41598-019-56167-7 (PMC6934560; doi:10.1038/s41598-019-56167-7)
Supplement: Supplementary file 1 — Supplementary material [file 41598_2019_56167_MOESM1_ESM.pdf]

Supplementary materials

Title: The effect of mindfulness training on extinction retention

Authors: Johannes Björkstrand, Daniela Schiller, Jian Li, Per Davidson, Jörgen Rosén, Johan Mårtensson, Ulrich Kirk

Supplementary Tables and Figures

Table S1. *Displaying results of Shapiro-Wilks test for all outcome measures investigating whether assumptions of normality where met.*

	W	p
Pre BAI	0.979	0.818
Post BAI	0.972	0.623
BAI change score	0.969	0.545
Pre BDI	0.908	0.016
Post BDI	0.941	0.110
BDI change score	0.949	0.172
Pre MAAS	0.960	0.325
Post MAAS	0.958	0.286
MAAS change score	0.979	0.824
Acquisition: Mean CS difference score	0.966	0.520
Extinction: Mean CS difference score	0.962	0.401
Extinction: Last trial CS difference score	0.763	< .001
Re-extinction: First trial CS difference score	0.930	0.068
Spontaneous recovery index (SRI)	0.960	0.394

Note. Significant results suggest a deviation from normality.

Table S2. *Displaying results of between group analysis (independent t-tests) before and after treatment as well as change scores for self-reported depression and anxiety symptoms.*

	t	df	p
Pre BAI	0.341	27.000	0.736
Post BAI	-0.668	27.000	0.509
BAI change score	-1.013	27.000	0.320
Pre BDI	-0.345	27.000	0.733
Post BDI	0.404	27.000	0.690
BDI change score	0.670	27.000	0.508

Table S3. *Descriptive statistics for the BAI, BDI and MAAS for each group separately, before and after treatment as well as changes-score.*

	Group	N	Mean	SD	SE
Pre BAI	Mindfulness	14	11.500	4.346	1.161
	Waitlist	15	11.000	3.546	0.915
Post BAI	Mindfulness	14	10.643	3.028	0.809
	Waitlist	15	11.400	3.066	0.792
BAI change score	Mindfulness	14	-0.857	3.394	0.907
	Waitlist	15	0.400	3.291	0.850
Pre BDI	Mindfulness	14	4.929	2.495	0.667
	Waitlist	15	5.267	2.764	0.714
Post BDI	Mindfulness	14	4.857	1.167	0.312
	Waitlist	15	4.600	2.098	0.542
BDI change score	Mindfulness	14	-0.071	2.235	0.597
	Waitlist	15	-0.667	2.526	0.652
Pre MAAS	Mindfulness	14	55.929	6.639	1.774
	Waitlist	15	56.133	14.227	3.673
Post MAAS	Mindfulness	14	62.000	8.171	2.184
	Waitlist	15	56.333	8.772	2.265
MAAS change score	Mindfulness	14	6.071	4.615	1.234
	Waitlist	15	0.200	10.455	2.700

Table S4. *Descriptive statistics for the SCR-based outcome measures.*

	Group	N	Mean	SD	SE
Acquisition: Mean CS differences score	Mindfulness	11	0.095	0.092	0.028
	Waitlist	15	0.086	0.117	0.030
Extinction: Mean CS difference score	Mindfulness	11	0.010	0.039	0.012
	Waitlist	15	0.021	0.075	0.019
Extinction: Last trial CS difference score	Mindfulness	11	0.058	0.140	0.042
	Waitlist	15	0.024	0.189	0.049
Re-extinction: First trial CS difference score	Mindfulness	11	-0.030	0.429	0.129
	Waitlist	15	0.292	0.271	0.070
Spontaneous Recovery Index (SRI)	Mindfulness	11	-0.088	0.405	0.122
	Waitlist	15	0.268	0.329	0.085

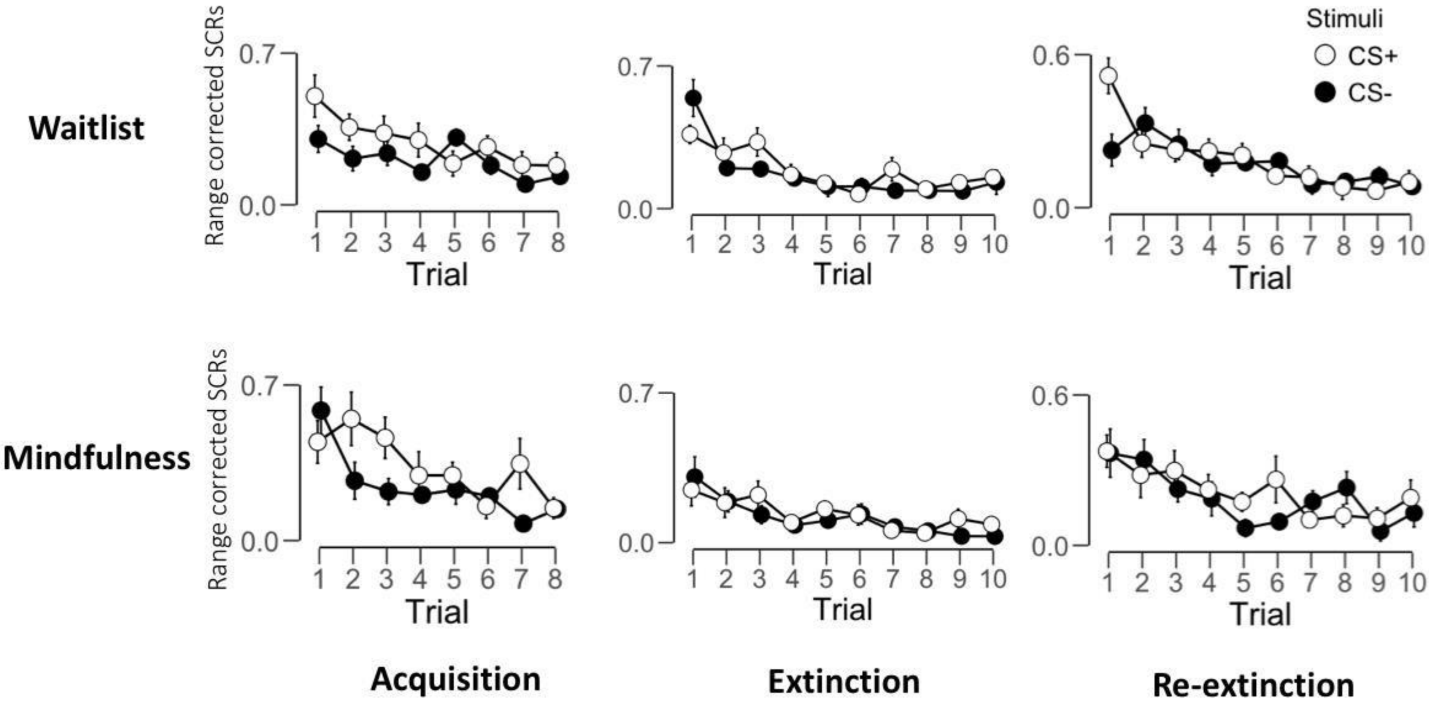

Figure S1. *Analysis of averaged CS difference scores did not reveal any significant group differences during either the acquisition or the extinction phase, but only a significant group effect for spontaneous recovery (see main text). In order to further explore the effect of MFT on fear and extinction learning and to give the reader a clearer picture of the data, we also performed trial by trial analysis for CS+ and CS- separately, for each of the experimental phases. For acquisition, single trial CS+ and CS- were entered into a 2x8x2 repeated measures ANOVA with factors Stimuli (CS+; CS-), Trial (1-8) and Group (Waitlist; Mindfulness). The results showed a significant main effect of Stimuli (F=17.89; p<.001), a significant main effect of Trial (F=10.69; p<.001), but no main effect of Group (F=1.14; p=.297), and no Trial x Group (F=0.91; p=.500), Stimuli x Trial (F=1.54; p=.157) or Stimuli x Trial x Group interaction (F=1.67; p=.120), see left column. The results are in line with analysis of averaged CS-difference scores, demonstrating successful acquisition of conditioned responses, but no group differences. Similarly, extinction was analyzed with a 2x10x2 repeated measures ANOVA with factors Stimuli (CS+; CS-), Trial (1-10) and Group (Waitlist; Mindfulness) which showed a significant main effect of Trial (F=12.27; p<.001), but no main effect of Stimuli (F=2.95; p=.098), or Group (F=1.79; p=.192), and no Trial x Group (F=.1.34; p=.218), Stimuli x Trial (F=1.87; p=.058) or Stimuli x Trial x Group interaction (F=0.59; p=.804), see middle column. The results are in line with analysis of averaged CS-difference scores, showing no significant difference between CS+ and CS- reactions and no group differences. In the same way re-extinction was analyzed, showing a significant main effect of Trial (F=10.51; p<.001), but no main effect of Stimuli (F=1.49; p=.233) or Group (F=0.17; p=.688) and no Trial x Group (F=0.58; p=.815) or Stimuli x Trial interaction (F=1.68; p=.095). There was however a significant Stimuli x Trial x Group interaction (F=1.92; p=.050), see right column. Post-hoc analysis of simple main effects indicates that the interaction effect was driven by differential responding to the CS+ and CS- on the first re-extinction trial, where within the waitlist group we observed a large effect of Stimuli (F=17.41; p<.001) but no effect in the MFT group (F=0.00; p=.950). This is in line with the analysis for spontaneous recovery (see main paper) and indicates that the effect of MFT on extinction retention thus appears to be restricted to the initial response during re-extinction.*
